# Soluble intercellular adhesion molecule-1 for stable and acute phases of idiopathic pulmonary fibrosis

**DOI:** 10.1186/s40064-015-1455-z

**Published:** 2015-10-31

**Authors:** Ryo Okuda, Hidekazu Matsushima, Kazutetsu Aoshiba, Tomohiro Oba, Rie Kawabe, Koujiro Honda, Masako Amano

**Affiliations:** Department of Respiratory Medicine, Saitama Red Cross Hospital, 8-3-33 Kami-ochiai, Chuo-ku, Saitama, 338-8553 Japan; Department of Respiratory Medicine, Tokyo Medical University Ibaraki Medical Center, 3-20-1 Chuou, Ami, Inashiki, Ibaraki Japan

**Keywords:** ICAM-1, Idiopathic pulmonary fibrosis, Acute exacerbation, KL-6, SP-D

## Abstract

The levels of soluble intercellular adhesion molecule-1 (sICAM-1) have been reported to increase in patients with idiopathic pulmonary fibrosis. However, the utility of sICAM-1 has not been reported in detail. The aim of this study was to investigate whether sICAM-1 was a useful biomarker for stable idiopathic pulmonary fibrosis (IPF) and early phase of acute exacerbation of IPF. The patients who were diagnosed with IPF between 2013 and 2015 were enrolled. The levels of sICAM-1 and other interstitial pneumonia markers were measured. In this study, 30 patients with stable IPF and 11 patients with acute exacerbation of IPF were collected. Mean sICAM-1 levels were 434 ± 139 ng/mL for the stable phase of IPF, 645 ± 247 ng/mL for early phase of acute exacerbation of IPF, 534 ± 223 ng/mL for connective tissue disease-associated interstitial pneumonia, 221 ± 42 for chronic obstructive pulmonary disease, and 150 ± 32 ng/mL in healthy volunteers. For the stable phase of IPF, sICAM-1 levels correlated with Krebs von den Lungen-6 (KL-6) (r value: 0.41; p value: 0.036). Mean sICAM-1 levels were significantly higher in patients with early phase of acute exacerbation of IPF than with stable phase of IPF (p = 0.0199). Multiple logistic analyses indicated that the predictors for early phase of acute exacerbation of IPF were only sICAM-1 and C-reactive protein (odds ratio: 1.0093; 1.6069). In patients with stable IPF, sICAM-1 levels correlated with KL-6; sICAM-1 might be a predictive indicator for prognosis. In the early phase of acute exacerbation of IPF, sICAM-1 might be more useful for diagnosis than other interstitial pneumonia markers.

## Background

Intercellular adhesion molecule-1 (ICAM-1) is a glycoprotein with a molecular weight of 80–110 kD belonging to the immunoglobulin superfamily. Stimulation of inflammatory cytokines such as interferon and interleukin (IL)-1 leads to expression of ICAM-1 in vascular endothelial cells, tracheal epithelial cells, and fibroblasts within a few hours (Munro et al. 1989; Hubbard and Rothlein 2000; Vogetseder et al. 1989). As the expressed ICAM-1 is involved in adhesion to white blood cells (WBCs), it is considered to be a protein involved early in the immune response (Albelda et al. 1994). As some of the expressed ICAM-1 is released into the circulating blood, measurement of soluble ICAM-1 (sICAM-1) levels in blood makes it possible to estimate ICAM-1 expression in the tissue.

For example, sICAM-1 levels have been reported to be increased in many inflammatory diseases such as infections, autoimmune diseases, and allergic diseases (Greve et al. 1989; Davies et al. 1992; Wegner et al. 1990). Furthermore, sICAM-1 levels have been reported to increase in pulmonary diseases, including chronic obstructive pulmonary disease (COPD), idiopathic pulmonary fibrosis (IPF), and connective tissue disease-associated interstitial pneumonia (CTD-associated IP) (Risse et al. 1994; Richards et al. 2012; Hasegawa et al. 2014). Few studies have compared sICAM-1 levels in different pulmonary diseases. Although Krebs von den Lungen-6 (KL-6) and surfactant protein D (SP-D) are used clinically in Japan as prognostic markers for IPF (Yokoyama et al. 2006; Takahashi et al. 2000), few studies have investigated the relationship between these and sICAM-1 in patients with IPF. We investigated these questions in this study.

Acute exacerbation of IPF is a disease with a poor prognosis in which new ground-glass opacities and infiltrates in both lungs and rapid respiratory failure appear in patients with chronic IPF (Kondoh et al. 1993). The Japanese guidelines state that blood KL-6, SP-D, C-reactive protein (CRP), and lactate dehydrogenase (LDH) are reference markers for acute exacerbation of IPF. As sICAM-1 levels increase in the early stages of inflammation, we estimated that sICAM-1 could become a biomarker for early phase of acute exacerbation of IPF.

## Methods

### Subjects

The patients who were diagnosed with IPF, CTD-associated IP, and COPD at Saitama Red Cross Hospital between July 2013 and July 2015 were enrolled in this study. IPF was diagnosed according to the following criteria: The patient was 50 years or older and conformed with the 2011 American Thoracic Society/European Respiratory Society IPF statement (Raghu et al. 2011); high-resolution computed tomography (CT) images usually showed usual interstitial pneumonia (UIP) or possible UIP, and UIP caused by collagen disease, hypersensitivity pneumonitis, or drug-induced pneumonia was clinically excluded. For CTD-associated IP, two collagen disease specialists and two respiratory disease specialists at Saitama Red Cross Hospital had diagnosed CTD-associated IP based on physical findings, blood tests, and high-resolution CT images.

“Stable IPF” was defined as patients with IPF who are stable for 2 months before the examination. In stable IPF, sICAM-1, KL-6 (reference range 0–500 U/mL), SP-D (reference range 0–110 ng/mL), CRP (reference range 0–0.5 mg/dL), LDH (reference range 119–229 UI/L), and WBCs (reference range 3100–8800/μL) were measured. Similarly, sICAM-1 was measured in healthy volunteers and patients with CTD-associated IP and COPD.

The diagnosis of acute exacerbation of IPF was based on the Japanese guidelines. Hence, after excluding other diseases such as obvious infections or heart failure, acute exacerbation of IPF was diagnosed based on the presence of all of the following: Increased severity of dyspnea, high-resolution CT findings showed honeycomb lung and new ground-glass appearances and infiltrates, and a decrease in partial pressure of oxygen in arterial blood of 10 mmHg or greater. On the day when acute exacerbation of IPF was diagnosed, blood sICAM-1, KL-6, SP-D, et al. were measured. Patients for which blood was collected on the first day of acute exacerbation of IPF were defined as “AEx IPF”. Almost AEx IPF cases underwent repeat measurements of sICAM-1 levels in the stable phase before or after acute exacerbation of IPF. All the blood samples in this study were centrifuged after clot formation. The serum samples were stored at −20 °C prior to analysis. Serum levels of ICAM-1 were measured by enzyme-linked immunosorbent assay (SRL Inc., Tokyo, Japan). This study was approved by the institutional review board of the Saitama Red Cross Hospital. Written consent was received from all patients.

### Immunohistochemical and immunofluorescence staining

Lung tissue samples were taken during surgery or autopsy performed at Saitama Red Cross Hospital in the past. Non-cancerous lung tissue samples were selected (two cases in each group) from patients of lung cancer with stable IPF, CTD-associated IP, and COPD receiving lung surgery. Autopsy lung tissue samples from AEx IPF cases (two cases) were used. AEx IPF and COPD lung tissue was taken from patients different from those in which serum sICAM-1 levels were measured. Resected tissue was embedded in paraffin after immersion fixation for 4 h in 10 % formalin. Lung tissue was cut into 3 μm-thick slices. The use of the tissue samples was approved by the ethical review board of our hospital.

Lung tissue underwent immunohistochemical staining using a fully automated immunohistochemical stainer (Ventana BenchMark GX, Ultra View DAB: Roche Diagnostics International Ltd., Tokyo, Japan). For antigen activation, specimens underwent heat processing (95–100 °C) for 60 min in ethylenediaminetetraacetic acid buffer solution (pH 8.5) and we used rabbit polyclonal antibody against intracellular adhesion molecule-1 (Santa Cruz Biotechnology INC., Texas, the USA: 1:4000 dilution) as the primary antibody. For the horseradish peroxidase reaction chromogenic substrate, 3,3′-diaminobenzidine and compared nuclear staining with hematoxylin were used.

Lung tissue also underwent manual immunofluorescence staining. Paraffin-embedded lung tissue was deparaffinized, hydrated and autoclaved in citrate acid buffer (pH 6.0, 121 °C for 15 min). Bovine serum albumin (3 %) was used to block non-specific hydrophobic binding for 30 min and we used rabbit polyclonal antibody against ICAM-1 (1:400 dilution) as the primary antibody for 1 h. Secondary antibody with Alexa Flour 488-conjugated anti-rabbit IgG (Abcam Plc., Cambridge, UK) and counterstain with DAPI were used.

### Statistical analysis

The unpaired t test was used for comparisons between two groups. To investigate the correlation between sICAM-1 and other interstitial pneumonia markers, Spearman’s rank correlation coefficient was used. Items suspected to be related to early acute exacerbation (sICAM-1, KL-6, SP-D, LDH, CRP, and WBC) were evaluated using univariate and multivariate logistic regression analyses. The analysis was performed with the Excel Statistics software (SSRI Co., Ltd.).

## Results

**Fig. 1:**
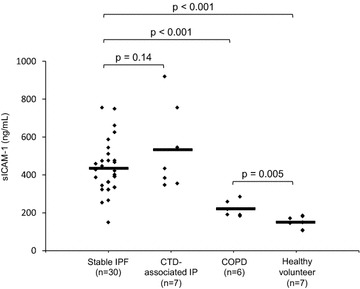
Levels of sICAM-1 in healthy volunteers and patients with Stable IPF, CTD-associated IP, and COPD. *Black lines* represent the average values. The unpaired t test was used. *sICAM-1* soluble intercellular adhesion molecule-1, *IPF* idiopathic pulmonary fibrosis, *CTD-associated IP* connective tissue disease-associated interstitial pneumonia, *COPD* chronic obstructive pulmonary disease

For the 30 patients with stable IPF, mean percentage forced expiratory vital capacity (%FVC) was 78.4 % and percentage diffusing capacity of the lungs for carbon monoxide (%DLco) was 74.9 %, indicating many patients with mild IPF (Table 1). There were seven patients with CTD-associated IP, six patients with COPD, and seven healthy volunteers. Mean sICAM-1 levels were 432 ± 139 ng/mL for stable IPF, 534 ± 223 for CTD-associated IP, 221 ± 42 ng/mL for COPD and 150 ± 32 ng/mL for healthy volunteers. Thus, sICAM-1 levels were significantly higher in patients with COPD than in healthy volunteers. In patients with stable IPF and CTD-associated IP, sICAM-1 levels were significantly higher than those in healthy volunteers and patients with COPD (Fig. 1). Investigation of possible correlations between biomarkers in patients with stable IPF indicated that sICAM-1 levels exhibited a positive correlation with KL-6 and CRP (r value 0.41; 0.36) (Table 2).

**Table 1:** Characteristics of study population

Characteristics	Stable IPF	CTD-associated IP	COPD	Healthy volunteer
Subjects	30	7	6	7
Male/female	25/5	3/4*	6/0	1/6**
Age (years)	72.3 ± 6.3	73.3 ± 6.8	75.5 ± 6.5	26.9 ± 4.0**
Smoking history				
Never/Ex- and current	7/23	3/4	0/6	6/1**
Treatment received				
None	12	0	N/A	N/A
N-acetylcysteine	9	0	N/A	N/A
Pirfenidone	2	0	N/A	N/A
Steroids	3	0	N/A	N/A
Steroids + immunosuppressant	3	5	N/A	N/A
Steroids + Pirfenidone	1	2	N/A	N/A
Blood tests				
KL-6 (U/mL)	1174 ± 1008	951 ± 544	N/A	N/A
SP-D (ng/mL)	240 ± 131	218 ± 215	N/A	N/A
WBC (/μL)	7660 ± 2290	11000 ± 2290**	9270 ± 2990	N/A
LDH (IU/L)	233 ± 58	246 ± 55	173 ± 34*	N/A
CRP (mg/dL)	1.00 ± 2.49	2.00 ± 2.87	0.8 ± 1.08	N/A
Pulmonary function				
VC % pred (%)	78.6 ± 23.4	75.5 ± 14.6	74.5 ± 13.9	N/A
FVC % pred (%)	78.4 ± 23.1	74.1 ± 16.2	70.0 ± 11.3	N/A
FEV_1_ % pred (%)	99.8 ± 28.1	86.7 ± 26.7	38.9 ± 11.7**	N/A
FEV_1_/FVC (%)	88.1 ± 6.2	80.0 ± 11.4	37.8 ± 8.1**	N/A
DLco % pred (%)	74.9 ± 28.9	56.7 ± 19.0	94.1 ± 34.5	N/A

Data are presented as n or mean ± standard deviation, unless otherwise stated. The unpaired t test was used (versus stable IPF)

*IPF* idiopathic pulmonary fibrosis, *CTD-associtated IP* connective tissue disease-associated interstitial pneumonia, *COPD* chronic obstructive pulmonary disease, *N/A* not available, *KL-6* Krebs von den Lungen-6, *SP-D* surfactant protein D, *WBC* white blood cell, *LDH* lactate dehydrogenase, *CRP* C-reactive protein, *VC* vital capacity, % *pred*  % predicted, *FVC* forced vital capacity, *DLco* diffusion capacity of the lung for carbon monoxide

* p < 0.05, ** p < 0.01

**Table 2:** Correlations between sICAM-1 and other parameters in patients with stable IPF

Parameters	p value	r value
KL-6	0.036	0.41
SP-D	0.15	0.27
LDH	0.76	0.06
CRP	0.0496	0.36
FVC % pred	0.25	−0.22
DLco % pred	0.18	−0.27

Spearman’s rank correlation coefficient was used

*sICAM-1* soluble intercellular adhesion molecule-1, *IPF* idiopathic pulmonary fibrosis, *KL-6* Krebs von den Lungen-6, *SP-D* surfactant protein D, *LDH* lactate dehydrogenase, *CRP* C-reactive protein, *FVC* forced vital capacity, % *pred* % predicted, *DLco* diffusion capacity of the lung for carbon monoxide

**Fig. 2:**
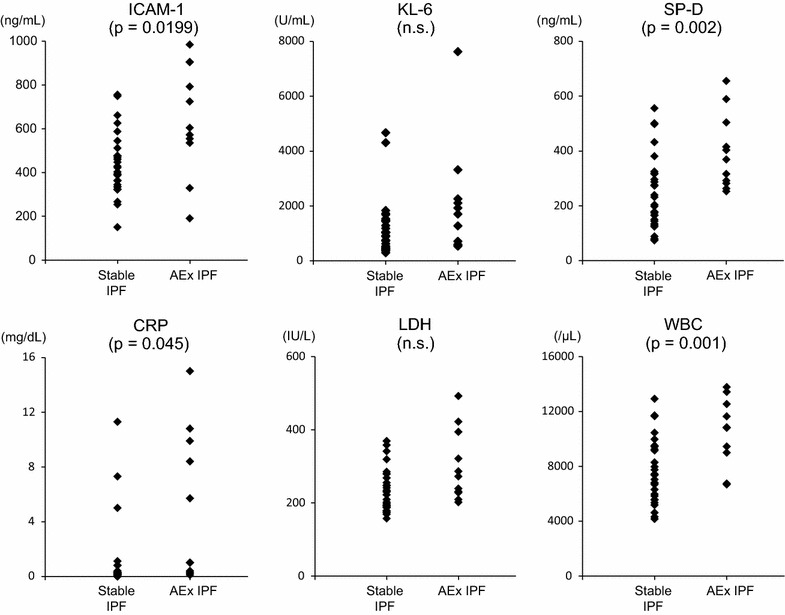
Levels of biomarkers in patients with Stable IPF and AEx IPF. The unpaired t test was used. *sICAM-1* soluble intercellular adhesion molecule-1, *KL-6* Krebs von den Lungen-6, *SP-D* surfactant protein D, *CRP* C-reactive protein, *LDH* lactate dehydrogenase, *WBC* white blood cell

**Fig. 3:**
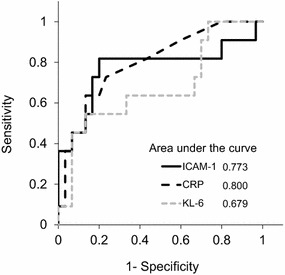
Receiver operator characteristics curves for identification of patients with acute exacerbation of idiopathic pulmonary fibrosis. *sICAM-1* soluble intercellular adhesion molecule-1, *KL-6* Krebs von den Lungen-6, *SP-D* surfactant protein D

Among the 11 patients with AEx IPF, mean sICAM-1 levels were 645 ± 247. Mean sICAM-1 levels were significantly higher in cases with AEx IPF than with stable IPF (p = 0.0199) (Fig. 2). Investigation of possible correlations between biomarkers in patients with AEx IPF indicated that sICAM-1 levels exhibited a positive correlation with only KL-6 (r value 0.82) (Table 3). Comparison of biomarkers in cases with AEx IPF and stable IPF indicated that independent predictors for early phase of acute exacerbation of IPF were sICAM-1 and CRP (Table 4). The optimal sICAM-1 cutoff for AEx IPF and stable IPF cases was found to be 535 ng/mL by receiver operating characteristic curve analysis (Fig. 3).

**Table 3:** Correlations between sICAM-1 and other parameters in patients with early phase of acute exacerbation of IPF

Parameters	p value	r value
KL-6	0.002	0.82
SP-D	0.73	0.12
LDH	0.63	0.16
CRP	0.017	−0.70
WBC	0.25	−0.38

Spearman’s rank correlation coefficient was used

*sICAM-1* soluble intercellular adhesion molecule-1, *IPF* idiopathic pulmonary fibrosis, *KL-6* Krebs von den Lungen-6, *SP-D* surfactant protein D, *LDH* lactate dehydrogenase, *CRP* C-reactive protein, *WBC* white blood cell

**Table 4:** Risk factors of early phase of acute exacerbation of IPF

Parameters	p value	Odd ratio	95 % CI
Univariate regression analysis			
sICAM-1	0.007	1.0062	1.0017–1.0107
KL-6	0.114	1.0004	0.9999–1.0010
SP-D	0.008	1.0079	1.0020–1.0137
LDH	0.024	1.0120	1.0016–1.0225
CRP	0.015	1.2831	1.0459–1.5696
WBC	0.006	1.0005	1.0001–1.0009
Multivariate logistic regression analysis			
sICAM-1	0.028	1.0093	1.0010–1.0176
CRP	0.009	1.6069	1.1248–2.2957

*IPF* idiopathic pulmonary fibrosis, *CI* confidence interval, *sICAM-1* soluble intercellular adhesion molecule-1, *KL-6* Krebs von den Lungen-6, *SP-D* surfactant protein D, *LDH* lactate dehydrogenase, *CRP* C-reactive protein, *WBC* white blood cell

### Lung histopathology

**Fig. 4:**
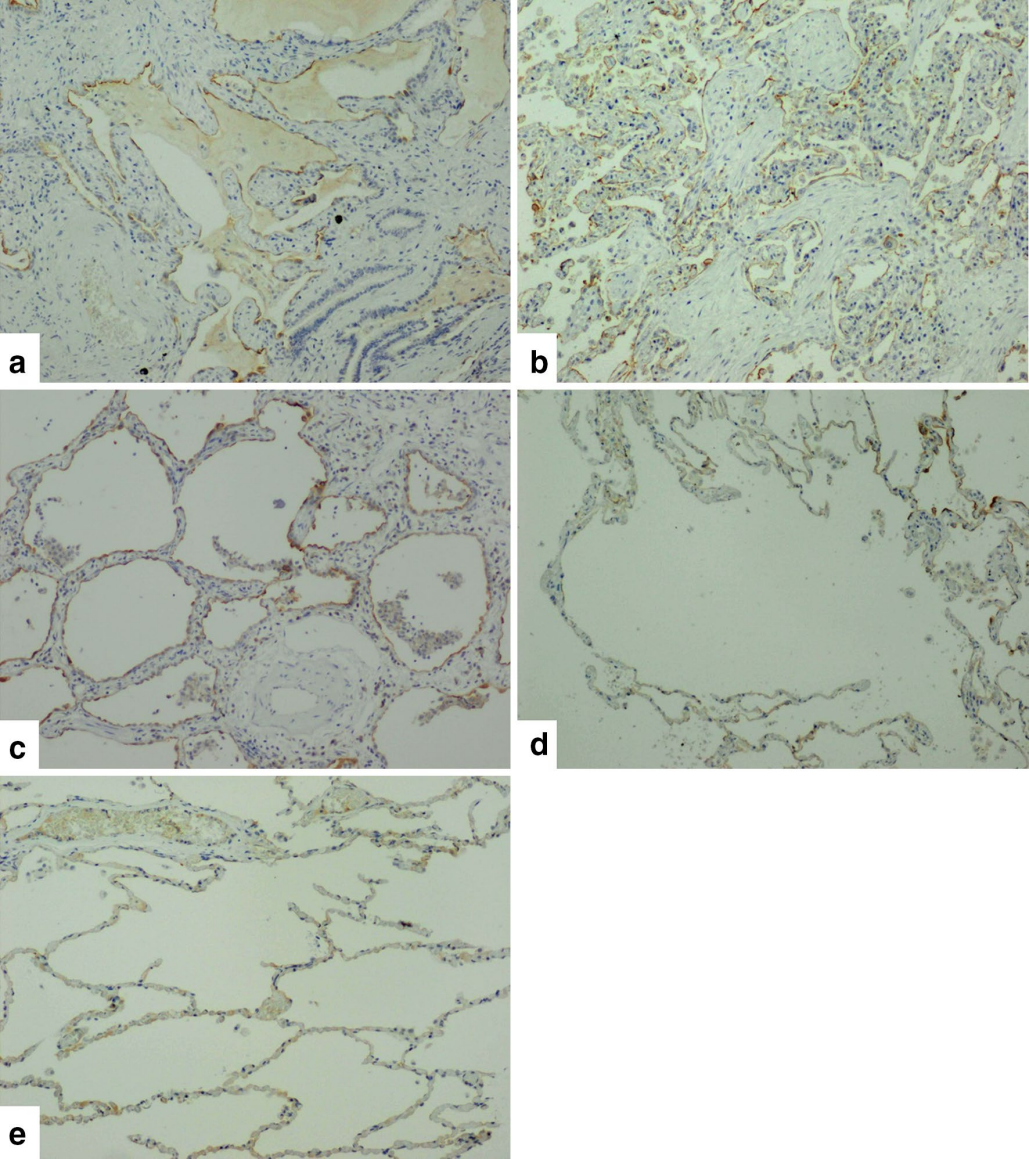
Immunohistochemical staining for ICAM-1 in the lung tissue. Contrast nuclear staining with hematoxylin was used. Stable IPF (**a**), AEx IPF (**b**), CTD-associated IP (**c**), COPD (**d**), normal lung (**e**). Original magnifications, ×10

**Fig. 5:**
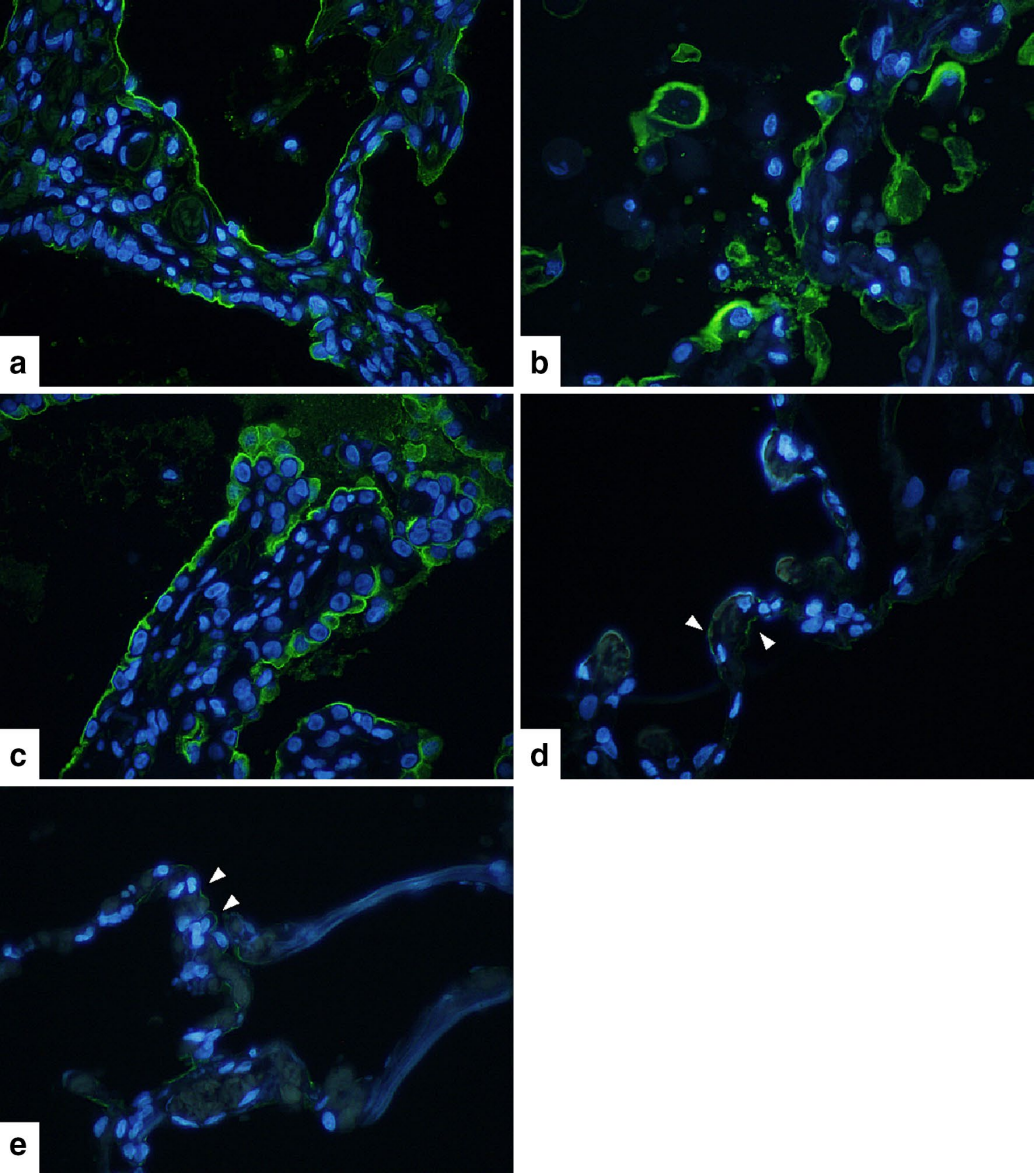
Immunofluorescence staining for ICAM-1 (*green*). Contrast nuclear staining with DAPI (*blue*) was used. Stable IPF (**a**), AEx IPF (**b**), CTD-associated IP (**c**), COPD (**d**), normal lung (**e**). *Arrowheads* pointed to examples of ICAM-1. Original magnifications, ×40

ICAM-1 in lung tissue was investigated using immunohistochemical and immunofluorescence staining. In healthy tissue, ICAM-1 was rarely expressed; however, in patients with stable IPF, ICAM-1 was observed, particularly, in the epithelial cells of cysts and bronchiectasis. ICAM-1 was also observed in some vascular endothelial cells. In cases with CTD-associated IP and AEx IPF, ICAM-1 expression was even stronger than that observed in cases with stable IPF (Figs. 4, 5).

## Discussion

This study demonstrated that sICAM-1 levels were increased in patients with stable IPF and that a positive correlation existed between sICAM-1 and KL-6. This study also indicated that sICAM-1 levels were more useful than KL-6 or SP-D for diagnosing the early phase of acute exacerbation of IPF.

It has previously been reported that sICAM-1 levels are elevated in diseases other than IPF (Greve et al. 1989; Davies et al. 1992; Wegner et al. 1990), and sICAM-1 levels were thought to have little disease specificity to IPF. Few studies have compared sICAM-1 levels in patients with IPF and COPD. In this study, sICAM-1 levels were significantly higher in cases with stable IPF and CTD-associated IP than in cases with COPD. When compared to previous reports on IPF (Takehara et al. 2001; Tsoutsou et al. 2004) and COPD (Oelsner et al. 2013) related to sICAM-1, sICAM-1 levels were higher in patients with IPF than COPD. Differences in ICAM-1 expression in lung tissue were also observed between patients with IPF and COPD in this study, which was consistent with the biomarker results.

Few studies have compared sICAM-1 levels with other interstitial pneumonia markers such as KL-6. In this study, sICAM-1 levels correlated with KL-6 in the stable phase. As KL-6 is a strong prognostic marker of IPF (Yokoyama et al. 2006), sICAM-1 levels might be a useful prognostic indicator. A previous study also reported that sICAM-1 levels could be a prognostic marker of IPF (Richards et al. 2012). Meanwhile, no correlation was observed between sICAM-1 and SP-D. The phenomenon of dissociation of KL-6 and SP-D has previously been indicated. It has been reported that KL-6 tends to strongly reflect honeycomb lung features, whereas SP-D reflects ground-glass opacities on high-resolution CT (Takahashi et al. 2000). In this study, immunohistochemical staining of stable IPF cases demonstrated a higher degree of ICAM-1 staining in the honeycomb lung or epithelial cells of bronchiectasis than in interstitial cells. Thus, sICAM-1 levels appear to be a biomarker that is more similar to KL-6 than SP-D.

Acute exacerbation of IPF, which occurs in approximately 5–15 % of cases with stable IPF per year, has a poor prognosis with a mortality rate of 50–80 % (Song et al. 2011). Early diagnosis is therefore considered crucial. KL-6 has been widely investigated in the markers of acute exacerbation of IPF (Zhang and Kaminski 2012). It has been reported that KL-6 levels rise in patients with acute exacerbation of IPF (Collard et al. 2010; Ishikawa et al. 2012). On the other hand, KL-6 levels do not always rise in the early phase of acute exacerbation of IPF (Ishizaka et al. 2004; Kakugawa et al. 2013). In this study, sICAM-1 levels were elevated already on the first day diagnosed as acute exacerbation of IPF, suggesting that it could be a biomarker for early phase of acute exacerbation of IPF. Because the expression of ICAM-1 was enhanced in the initial phase of inflammation and early phase of acute respiratory distress syndrome (Schütte et al. 1996; Pugin et al. 1996; Kuppner et al. 1990), sICAM-1 levels may also rise in the early phase of acute exacerbation of IPF.

The limitations of this study include the fact that prognosis could not be investigated due to the small sample size. A previous report demonstrated that sICAM-1 levels were a prognostic factor for IPF (Risse et al. 1994). Hence, verification with large samples of IPF and CTD-associated IP cases is required. Furthermore, the tissue samples used for acute exacerbation cases were from autopsy cases, and therefore, these were not tissue samples from the early phase of acute exacerbation. We also cannot rule out the influence of treatments such as steroid pulse therapy. Despite this, strong ICAM-1 staining in lung tissue was observed in cases with AEx IPF.

In conclusion, sICAM-1 levels in patients of stable IPF strongly correlated with KL-6; sICAM-1 might be a predictive indicator for prognosis. In the early phase of acute exacerbation of IPF, sICAM-1 might be more useful for diagnosis than other interstitial pneumonia markers. In stable and acute phases of IPF, sICAM-1 might be an important biomarker.

## Abbreviations

sICAM-1: soluble intercellular adhesion molecule-1
IPF: idiopathic pulmonary fibrosis
IL: interleukin
WBC: white blood cell
COPD: chronic obstructive pulmonary disease
CTD-associated IP: connective tissue disease-associated interstitial pneumonia
KL-6: Krebs von den Lungen-6
SP-D: surfactant protein D
CRP: C-reactive protein
LDH: lactate dehydrogenase
CT: computed tomography
UIP: usual interstitial pneumonia
%FVC: percentage forced expiratory vital capacity
%DLco: percentage diffusing capacity of the lungs for carbon monoxide
FEV_1_: forced expiratory volume in one second
